# Relationship Between Ankle Plantar Flexion Angle and Tendon Gap in Achilles Tendon Rupture: A Prospective Study Using Portable Handheld Ultrasonography

**DOI:** 10.7759/cureus.109845

**Published:** 2026-05-28

**Authors:** Gregory Waryasz, Atta Taseh, Nour Nassour, Noopur Ranganathan, Jonathan Kattady, John Kwon, Lorena Bejarano-Pineda, Christopher W DiGiovanni, Soheil Ashkani-Esfahani, Daniel Guss

**Affiliations:** 1 Foot and Ankle Research and Innovation Lab - FARIL, Department of Orthopaedic Surgery, Mass General Brigham, Harvard Medical School, Boston, USA; 2 Foot and Ankle Division, Department of Orthopaedic Surgery, Massachusetts General Hospital, Boston, USA

**Keywords:** achilles injury, foot and ankle examination, patient-reported outcomes, point of care imaging, ultrasonography

## Abstract

Background

Achilles tendon rupture (ATR) requires accurate diagnosis and timely intervention to reduce complications, disabilities, and costs. ATR gap influences treatment decisions, surgical or non-surgical. This study aimed to assess the correlation between ankle angle in knee flexion at 90 degrees and ATR gap size, exploring whether this method could reliably detect ATR and estimate the tendon gap.

Methods

In this prospective study, 47 patients with acute ATR underwent pre-operative assessment using portable handheld ultrasound. Measurements included the tendon gap at various foot positions and ankle angles in knee flexion. Baseline patient-reported outcome measures (PROMs) were also collected. Spearman's correlation was used to analyze relationships between gap size, angle measurements, and PROMs, while receiver operating characteristic (ROC) analysis identified an optimal ankle angle threshold.

Results

No significant correlation emerged between tendon gap size and angle values, nor between PROMs and angle or gap measurements. However, a significant difference in plantar flexion angles was found between injured and uninjured ankles, with a median of 99.4° for injured and 110.6° for healthy sides (p < 0.001). The optimal threshold of 104.75° achieved 83% sensitivity and 78% specificity in distinguishing ATR.

Conclusion

Ankle angle measurement may enhance ATR screening in settings without advanced imaging. The identified threshold could assist in clinical evaluation, though further research is needed to validate its use alongside portable handheld ultrasound as an accessible, cost-effective diagnostic tool.

## Introduction

The Achilles tendon is the largest and strongest tendon in the body, as well as the most frequently injured one [1]. The progressively aging population, coupled with the rise in obesity prevalence and increasing participation in sports, has all been mentioned to contribute to the increasing incidence of Achilles tendon ruptures (ATRs) [1-3]. Although recent studies have shown favorable outcomes with conservative management supplemented with functional rehabilitation, surgical intervention is typically recommended, as evidenced by the lower re-rupture rates [4-6]. ATR patients usually describe a popping sound in their leg or a feeling of being kicked in the posterior ankle. On physical examination, signs of ATR can include difficulty with weight bearing, limping, weakness of plantar flexion strength, a positive Thompson test, increased passive ankle dorsiflexion, and the presence of a palpable defect [1,7]. The diagnosis is typically supported with imaging assessments, such as magnetic resonance imaging (MRI) or ultrasonography, as confirmatory tests [8]. Despite nearly perfect accuracy, MRI is not frequently used due to its associated costs and the delayed time to diagnosis [9,10].

On the other hand, ultrasonography has shown comparative utility in recognizing gaps in ATRs [1]. Recent advances in portable handheld ultrasound technology have increased accessibility and reduced cost while avoiding ionizing radiation [11-13]. This can expedite the diagnosis process and facilitate timely intervention and improved outcomes in ATR [14]. Various studies have shown that a greater ATR gap found in ultrasonography (> 5-10 mm) correlates with poorer outcomes and a higher risk of re-rupture [15-17]. The dynamic nature of ultrasound allows for ankle dorsiflexion and plantarflexion as part of the examination [18]. This helps in differentiating between partial and complete ruptures, with an increase in gap during dorsiflexion being indicative of a full-thickness tear [8]. However, whether there is any correlation between the plantar flexion angle and the ATR gap has rarely been assessed.

The aim of this study was to evaluate the correlation between resting ankle plantar flexion angle and ATR gap size. Furthermore, the diagnostic utility of ankle angle measurements for diagnosing ATRs using portable handheld ultrasonography was assessed.

## Materials and methods

In this prospective clinical study, 47 patients with acute ATR were consented and enrolled. Patients were eligible for inclusion if they were aged 18 years or older and had a diagnosis of ATR scheduled for surgical treatment by expert foot and ankle surgeons. Prospective patients were excluded if they had any concomitant trauma and injuries, as well as a history of prior ipsilateral Achilles tendon surgery or injury. The protocol of this study was approved by the institutional review board (IRB no. 2021P000213). The ultrasound devices were FDA approved (Butterfly IQ+™; Butterfly Network Inc., Burlington, MA). Ultrasound examinations and tendon-gap measurements were performed by board-certified foot and ankle surgeons with formal musculoskeletal ultrasound training and prior experience using ultrasound for Achilles tendon evaluation.

The injured side of the patient was examined using ultrasound in the operating room before the surgery started. The examination time was 5-15 minutes. We did not change the routine examination and treatment process of the patients, and the ultrasound examination was only an additional step to the routine care plan. Prior to the surgery, baseline functional status and symptoms were assessed using several validated patient-reported outcome measures (PROMs). Functional impairment to the Achilles tendon was assessed using the Achilles Tendon Total Rupture Score (ATRS), a questionnaire validated for monitoring recovery following tendon rupture [19]. General pain levels were quantified via a visual analogue scale (VAS) [20]. To characterize the impact of the injury on quality of life, we used the Patient-Reported Outcomes Measurement Information System (PROMIS) short forms for Pain Intensity and Pain Interference, which provide standardized metrics for physical and psychological symptom burden [21]. To conduct the examinations, the patient was placed in a prone position, and the gap length was measured using the ultrasound device in 3 foot positions: neutral at rest with extended knee, forced plantarflexion with extended knee, and neutral ankle with 90 degree knee flexion. Subsequently, the ankle angle was measured and compared in the injured and uninjured sides in 90 degrees of knee flexion using an electronic goniometer on the lateral aspects of the ankles. The proximal arm of the goniometer was aligned with the longitudinal axis of the fibula, and the distal arm was aligned with the lateral border of the foot. The ankle was allowed to rest passively without active patient contraction, and the same positioning technique was used for the injured and contralateral uninjured sides.

Ankle-angle measurements were performed by two experienced orthopaedic researchers under the direct supervision of the treating foot and ankle surgeon, who was present in the operating room and corroborated the measurement workflow. For each ankle-angle measurement, the measurement was performed twice to assess consistency. When the two measurements were consistent, one measurement was recorded for analysis. Plantar flexion was considered positive (> 90 degrees), and dorsiflexion was negative (< 90 degrees). Healthy ankles in neutral positions were slightly positive.

Statistical analysis

Using G Power software (V3.1, The G*Power Team, Germany) and based on the ankle angle values reported in a previous study by Zellers et al., we performed a sample size calculation to ensure adequate statistical power for our study [22]. Using a power of 90% and an alpha error rate of 0.05, we determined that a minimum of 39 participants would be required to detect significant differences. Accounting for a 20% dropout rate, the final sample size was determined to be 47. Baseline characteristics were evaluated using descriptive statistics, including the distribution of values, medians, and interquartile ranges (IQR). The relationship between ultrasound gap measurements and PROMs with ankle angle measurements was investigated using Spearman's rank correlation coefficient. Additionally, receiver operating characteristic (ROC) curve analysis was conducted. Using the area under the curve (AUC-ROC), the optimal cut-off points for ankle angle measurements that best distinguish between healthy and injured sides were established. Statistical significance was defined as p < 0.05. All analyses were performed using Statistical Product and Service Solutions (SPSS, version 28; IBM SPSS Statistics for Windows, Armonk, NY).

## Results

A total of 47 participants were enrolled in this study, consisting of 12.8% (6) females and 87.2% (41) males. The median age of participants was 37 years (IQR: 28-47).

**Figure 1:**
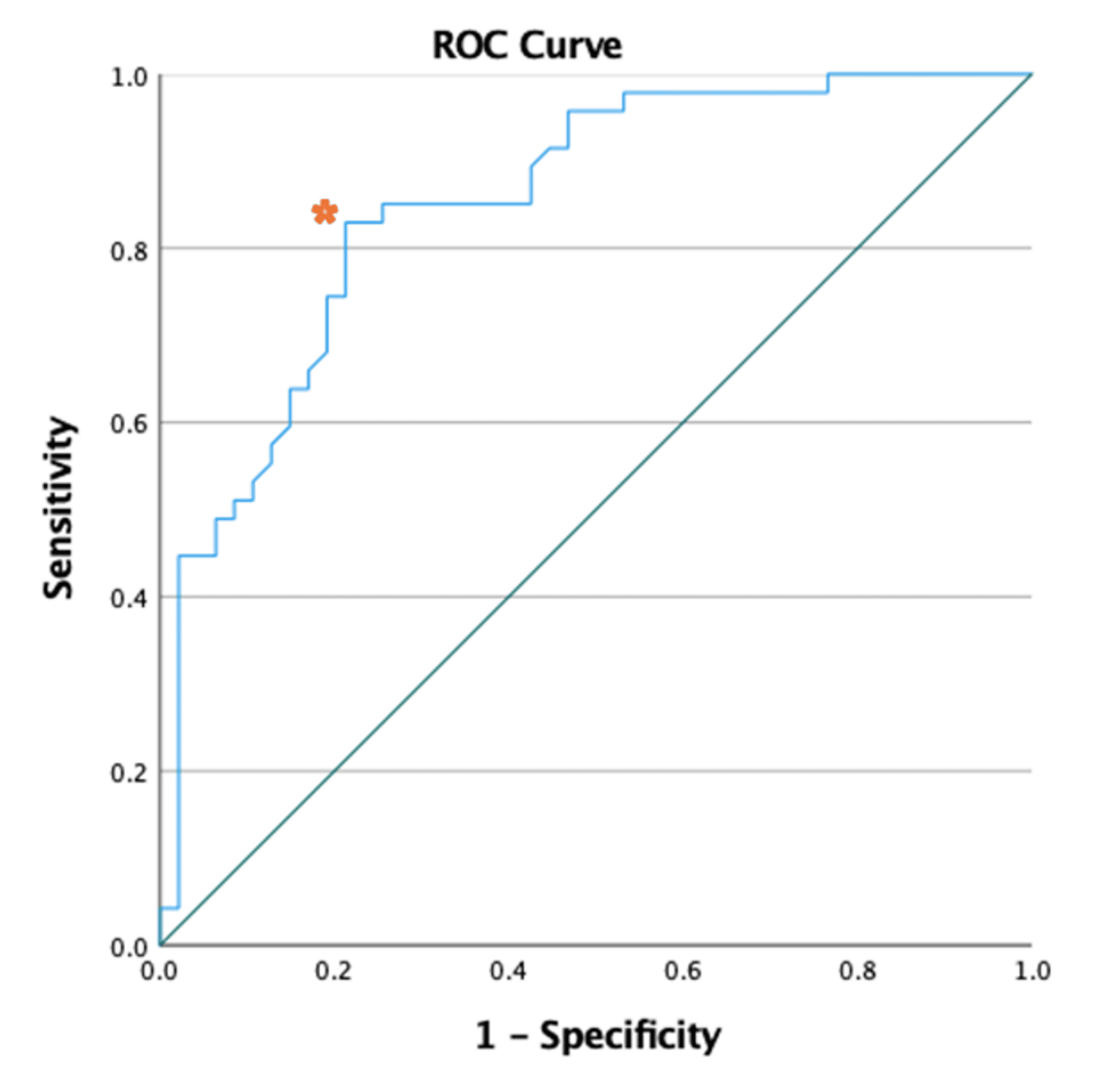
ROC Curve of the Resting Ankle Angle for the Diagnosis of ATR The ROC curve demonstrates the diagnostic accuracy of resting ankle angle measurements in differentiating between injured and uninjured ankles. The blue line represents the performance of the ankle angle as a diagnostic test. The orange asterisk (*) denotes the identified optimal threshold of 104.75° determined by the Youden Index, which corresponds to a sensitivity of 83% and a specificity of 78%. The diagonal reference line represents the line of identity, indicating a test with no diagnostic utility. ROC, Receiver Operating Characteristic; ATR, Achilles Tendon Rupture

No significant association was found between gap measurements and angle values (Table 1). Similarly, baseline PROMs did not demonstrate any significant correlations with either gap or angle measurements (Table 2). However, a statistically significant difference was observed in the angle of plantar flexion measurements between the injured and uninjured sides. The median angle on the injured side was 99.4 degrees (IQR: 93.6-103.3), whereas it was 110.6 degrees (IQR: 104.9-118.3) on the healthy side (p < 0.001). The optimal cut-off for differentiating between the injured and uninjured sides was determined to be < 104.75 degrees, providing a sensitivity of 83%, a specificity of 78%, and an AUC-ROC of 0.85. These findings are illustrated in Figure 1.

**Table 1:** Ultrasound Measurements of ATR Gap Size Across Various Clinical Positions and Their Correlation With Resting Ankle Angle *Spearman's rank correlation coefficient; p < 0.05 was considered significant. ATR, Achilles Tendon Rupture

Position	ATR Gap with Neutral Ankle	ATR gap with Ankle in Full Plantar Flexion	ATR Gap with Neutral Ankle + 90° Flexed Knee	Ankle Angle with Neutral Ankle + 90° Flexed Knee
Median (IQR)	1.5 (1.2-2.3)	0.8 (0.6-1.3)	1.4 (1.0-1.6)	99.4 (93.6-103.3)
Correlation Coefficient (p-value)*	0.90 (0.52)	0.18 (0.23)	0.15 (0.33)	-

**Table 2:** Baseline PROMs in ATR Patients and Their Correlation With Ankle Angle at 90° Knee Flexion and Neutral Ankle Position Ankle angle with neutral ankle + 90° flexed knee: median = 99.4° (IQR: 93.6-103.3°). †Spearman’s rank correlation coefficient; p < 0.05 was considered significant ATR, Achilles Tendon Rupture; ATRS, Achilles Tendon Total Rupture Score; IQR, Interquartile Range; PROM, Patient-Reported Outcome Measure; PROMIS, Patient-Reported Outcomes Measurement Information System; VAS, Visual Analog Scale

PROM	ATRS	PROMIS - Pain Intensity	PROMIS - Pain Interference	VAS Pain Score
Median (IQR)	85 (79-92)	54.2 (50-58)	63.1 (57-67)	2 (2-5)
Correlation Coefficient^†^	-0.01 (0.94)	-0.2 (0.17)	0.05 (0.72)	0.02 (0.90)

## Discussion

In this prospective study, we evaluated the diagnostic utility of ankle angle measurements for detecting ATR. Our findings highlight that resting ankle plantar flexion angle did not significantly correlate with ultrasound-measured ATR gap size. Additionally, plantar flexion angle differed significantly between injured and uninjured ankles, with a lower median angle on the injured side. ROC analysis identified an ankle-angle threshold of 104.75° for distinguishing injured from uninjured ankles, with 83% sensitivity, 78% specificity, and an AUC-ROC of 0.85. These findings suggest that ankle-angle measurement may have value as an adjunctive clinical observation, but this threshold should be considered exploratory and requires external validation before clinical use.

Reduced range of motion, especially plantarflexion of the foot, coupled with symptomatic pain, inability to bear weight, and reporting a popping sound upon forced dorsiflexion of the ankle, are all indicative signs of ATR [23]. The Thompson test has become one of the most sensitive clinical exams for diagnosing ATRs, with a reported sensitivity of 96%-100% and specificity of 93%-100%. The Matles test is another diagnostic test for ATR. By observing the position of the foot with the patient lying prone and knees bent at 90 degrees, a neutral or dorsiflexed foot indicates a possible rupture, while plantarflexion suggests an intact tendon [24]. The Copeland test is also very similar to the Thompson test and has a sensitivity of 79% [24]. The Copeland test may involve squeezing the calf at varying points to detect subtle or partial ruptures, providing a more detailed assessment. Other clinical measures on physical exam can also be helpful when trying to determine midportion rupture or insertional tear. For example, some studies note tendon stiffness as an important indicator of possible midportion Achilles tendinopathy, of which ATR is a subset [25,26]. Among all these tests, the Thompson test is favored for its simplicity, reliability, and high sensitivity, providing a clear indication of Achilles tendon integrity with minimal effort. In the present study, the ankle-angle threshold demonstrated lower sensitivity and specificity than reported values for the Thompson test. Therefore, ankle-angle measurement should not be considered a replacement for established physical examination maneuvers. Rather, it may serve as an adjunctive observation when examination findings are equivocal or when additional support is needed during initial assessment. Furthermore, it may inform future research aimed at developing diagnostic tests with improved specificity for partial tears.

Portable handheld ultrasound devices are proving to be highly effective for diagnosing Achilles tendon ruptures, offering notable advantages over MRI in terms of cost, accessibility, and usability. While MRI maintains high diagnostic standards with sensitivities and specificities around 95-100% for Achilles injuries, recent studies show that portable ultrasound can achieve accuracy rates of 90-96%, making it a reliable alternative in skilled hands [27,28]. In this cohort of surgically treated patients with clinically diagnosed ATR, portable handheld ultrasound identified the rupture site and allowed measurement of the tendon gap in all included cases. However, because the study did not include a non-ATR control group or nonoperative patients, diagnostic accuracy cannot be inferred from these data. Handheld ultrasound devices enable immediate, accessible assessments in various settings, such as sports clinics and emergency departments, supporting quick diagnosis and treatment planning [29]. However, they also remain operator-dependent, with image quality and interpretation influenced by probe positioning, anatomic knowledge, acquisition technique, and examiner experience. This raises the question of whether orthopedic surgeons and trainees should receive training in point-of-care ultrasound, allowing them to conduct assessments in outpatient settings, on the sports field, or even in military contexts, to provide swift care and alleviate the burden on the healthcare system [30].

Several limitations must be kept in mind for this study. First, although all ultrasound gap measurements were performed by board-certified foot and ankle surgeons with formal musculoskeletal ultrasound training and experience, formal interobserver or intraobserver reliability testing was not performed. Therefore, ICC values for continuous gap measurements were not calculated, and the reproducibility of the ultrasound gap measurements cannot be determined from the present study. Additionally, the presence of selection bias due to the inclusion of solely patients undergoing surgery makes it unclear if the findings would be different in conservatively managed patients. Similarly, although ankle-angle measurements were performed twice by two experienced orthopaedic researchers under surgeon supervision to ensure consistency, formal reliability statistics were not calculated for the goniometer measurements. Additionally, it is important to highlight that the majority of our population were males, which was expected due to the incidence of ATR; however, this lowers the generalizability of our outcomes and cut-off value. It is also important to note that all measurements were taken in an operating room by board-certified orthopedic surgeons with formal expertise in musculoskeletal ultrasound. As a result, these findings may not be generalizable due to the differences in examiner experience and the environment in emergency departments, outpatient clinics, athletic training rooms, or field settings. Finally, while we introduced a cut-off value based on AUC-ROC, our statistical analysis did not show a significant correlation between the Achilles gap and the ankle angle; the threshold should not be interpreted as a marker of rupture severity or tendon gap. This necessitates further studies on this topic to establish evidence on the possible role of ankle angle, especially for the more nuanced diagnostic task of distinguishing between partial vs complete ATRs.

## Conclusions

Our findings demonstrate that a measure - such as the ankle angle when the patient is prone and the knee is at 90 degrees - can be an added layer of confirmation in the physical examination process, allowing for an additional screening tool in the diagnosis of ATRs. However, this does not eliminate the need for further imaging modalities to confirm the diagnosis. The ankle angle method in a flexed knee can be used in settings where confirmatory imaging is not readily available, and there are no experts to assess for tendon gap or conduct the Thompson test. In order to assess the accuracy and generalizability of our cut-off value for ankle angle, further studies with larger and more diverse populations are suggested.
